# Supplementary material for: Dispositional and ideological factor correlate of conspiracy thinking and beliefs
Source: PLoS One. 2022 Oct 26;17(10):e0273763. doi: 10.1371/journal.pone.0273763 (PMC9604007; doi:10.1371/journal.pone.0273763)
Supplement: Attachment — Submitted filename: BDConspiracy comments to reviewers.docx [file pone.0273763.s002.docx]

**Subject: PLOS ONE Decision: Revision required [PONE-D-21-40074] - [EMID:2f14363b9625c539]**

**PONE-D-21-40074**

**Bright and Dark-Side Personality and Conspiracy Thinking**

**PLOS ONE**

Dear Dr. Knezevic

**Thank you for email concerning the above paper and attaching reviewers comments. I note below how we have responded to each.**

**Your Comments**

The hierarchical regression analysis of which results are presented in Table 9 is highly problematic. First of all, the way you had constructed dependent variable (factor with loadings on conspiracy variables, based on the factor analysis of the overall set of variables) was not appropriate. The reason is that it contains - to an extent – the variance of the variables that you were using as predictors later in this regression analysis (this is the reason why your multiple correlation is so inflated in this analysis). Moreover, the rationale for this analysis is totally unclear: you showed the results with conspiratorial thinking and conspiratorial beliefs separately (I see the logic in this – I guess to further check similarity of these two by projecting them in the space defined by the same predictor set), and then, you are showing practically the same thing with a variable that is some unfortunate composite of these two (and the rest of the variables included in this FA). I would highly recommend to remove this analysis, the text attached to it, as well as Figure 1.

**THIS PART IS NOW DELETED ENTIRELY**

A little can be achieved by stretching the notion of dark-of side personality on cluster A. Cluster A is implicated in conspiracy beliefs because it measures psychotic-like phenomena, not because of its darkness (if dark side of personality has anything in common with what is measured by dark triad or dark tetrad scales, i.e. malevolent personality). In other words, If you partial out schizotypy from Cluster A it would cease to predict anything conspiratorial (beliefs, thinking, mind-set). Cluster B (more related to the notion of dark personality) is obviously of secondary importance, even part of its relatedness to conspiracy is due to the correlation with Cluster A. Therefore, my recommendation would be to take into consideration the first suggestion of r#2 very seriously. I would recommend to rely on the usual use of the notion of darkness in this field. Your own results revealed much more peculiarity in conspiracy than darkness.

**THE DARKNESS PART IS NOW LARGELY DELETED FROM THE DISCUSSION AND THE FOCUS IS ON THE CLINICAL SCALES AND CLUSTERS.**

You did not find support neither for H2 nor H5. Please, be more careful with the interpretation of your own results.

**ALL HYPOTHESES ARE NOW RESTATED AND THEIR EMPIRICAL STATUS COMMENTED IN A WRAP-UP AT THE BEGINNING OF THE DISCUSSION**.

Your explanation of the mediator role of political ideology between conscientiousness and conspiracy does not seem to be convincing: openness and agreeableness are much more implicated in political ideology than conscientiousness.

**WE HAVE DELETED THIS PART OF THE ANALYSIS AND INSTEAD FOCUSED ON THE CONTRIBUTIONS OF THE PERSONALITY DISORDER CATEGORIES IN MULTIPLE REGRESSION.**

Variable "degree" is not explained (Years of schooling? Educational levels (if levels, how many)?). In regressions with CT1 and CT2 as Dvs, it looks like higher level of education predicts conspiracy, not the other way around. It is strange that your educational levels start with higher levels and end with lower levels (years) of education. Please, be careful with this - I suggest higher levels of this variable to reflect higher education level.

**WE PREVIOUSLY USED A REVERSE CODED, DICHOTOMOUS VARIABLE. NOW USING THE NUMBERS OF YEARS OF EDUCATION IN THE EXPECTED DIRECTION (HIGHER NUMBERS = MORE YEARS OF EDUCATION).**

Minor – rename Table 1 as Table 2.

**DONE AS REQUESTED**

Please, avoid using term interaction in describing your H9, you did not test the interaction between personality and ideology.

**NOW DELETED AS SUGGESTED**

Please, use the same labels throughout the article (e.g. BCTI but not CT2, or CT2 not BCTI, or use both).

**DONE AS REQUESTED**

**-------------------------------------------------------------------------------------------------------------**

When submitting your revision, we need you to address these additional requirements.

1. Please ensure that your manuscript meets PLOS ONE's style requirements, including those for file naming.

**HOPEFULLY THIS HAS BEEN DONE**

2. Please amend your current ethics statement to address the following concerns: a) Did participants provide their written or verbal informed consent to participate in this study? b) If consent was verbal, please explain i) why written consent was not obtained, ii) how you documented participant consent, and iii) whether the ethics committees/IRB approved this consent procedure.

**WE USED THE STANDARD PROCEDURE FOR PROLIFIC. THE ETHICS COMMITTEE CONSENTED TO PARTICIPANTS AGREEING TO HAVE THEIR ANONYMISED RESPONSES AGGREGATED, ANALYSED AND SUBSEQUENTLY PUBLISHED. WE HAVE DONE THIS WITH NUMEROUS STUDIES.**

2. We note that you have stated that you will provide repository information for your data at acceptance. Should your manuscript be accepted for publication, we will hold it until you provide the relevant accession numbers or DOIs necessary to access your data. If you wish to make changes to your Data Availability statement, please describe these changes in your cover letter and we will update your Data Availability statement to reflect the information you provide.

**WE CONSISTENTLY NOTE THAT IF ANYONE WISHES TO SEE THE DATA WE WILL SEND THEM AN SPSS FILE WITH THE VARIABLES EXPLAINED. THE FILE IS COMPLICATED AND HAS NUMBER OF VARIABLES THAT WE DID NOT ANALYSE OR REPORT FOR THIS STUDY**

3. Please include a copy of Table 5 which you refer to in your text on page 14. [Note: HTML markup is below. Please do not edit.]

**TABLES REVISED AND EDITED.**

**--------------------------------------------------------------------------------------------------------------**

Reviewer #1:

This paper presents findings from an online study of 397 community residents ages 19-71 most of whom were British in nationality. They completed an extensive battery of personality, political, and other demographic differences along with two measures of acceptance of conspiracies, a more general measure and one assessing belief in specific conspiracies. The two measures of conspiratorial thinking were moderately correlated (r=.58), which is about what one would expect given the respective levels of reliability (.60 and .91). Regression analyses using the various correlates produced similar profiles of associations, and a factor model placed the two measures in roughly the same location in a two-dimensional space, defined by political ideology versus measures of personality disorders.

**A FAIR SUMMARY**

The findings show that both types of measures of conspiratorial thinking are largely related in the same ways to various characteristics. There are more of those for the specific conspiracies, but that is not explained, and so I’m not sure there is much new here other than that. The authors suggest that they are testing the hypothesis that “personality and ideological beliefs interact to explain a common pathway toward CT.” (page 6). But I see no effort to test that hypothesis as all of the analyses involve tests of single correlates. There is evidence that after controlling for the personality disorders, the relation with political ideology disappears. But I don’t see how that tests the hypothesis as it is stated.

**WE BELIEVE THAT AS WE HAVE RESTATED THE HYPOTHESES AND REWRITTEN MUCH OF THE DISCUSSION WE HAVE NEW AND INTERESTING FINDINGS**

Apparently, persons with those disorders in this sample are more likely to be on the conservative end of the political spectrum. The authors claim that their findings support the prediction, but I don’t how they can say that other than that is what they are reading into the pattern of correlates.

**WE HAVE ATTEMPTED AN EXPLANATION OF THIS POINT**

The paper would benefit a lot from a more discerning discussion of what the findings say and what questions they answer.

**WE BELIEVE THAT WE HAVE NOW DONE THIS**

There is some confusion at the bottom of page 13, when the authors say that various characteristics were related to endorsement of CT theories: Extraverts with high scores on Cluster A and B, low on Cluster C were more likely to endorse CT theories.” But that is not what the regressions tell us. Those are independent predictors. And does CT include theories?

**THANK YOU. WE HAVE RE-VISTED AND CORRECTED THIS POINT**

Reviewer #2:

The paper is very well written, concise, and clear. The research problem is very relevant and actual and the strength of this study is in the involvement of different variables in the prediction of CT.

**EXCELLENT NEWS**

However, there are some concerns and suggestions for improvement of the manuscript:

- The title is not appropriate since the authors explored basic and psychopathological traits and not traits that are referred to the bright and dark side of personality in common sense (Dark Triad, Light Triad). Maybe title such is: Dispositional and ideological factors of conspiracy thinking and beliefs would be more appropriate.

**EXCELLENT SUGGESTION THANK YOU, NOW CHANGED**

- I am not sure what insight we get with separate analyses on CT1 and CT2. The final analysis presented in Table 9 seems that subsumed previous results and it would be economic to keep only these results since two conspiracy measures are already highly correlated. In the introduction, the authors did not explain differences between CT1 and CT2, nor their different correlates, thus separate analysis is not argumented. If they want to keep it (with more elaboration and arguments for that in Introduction), then in the 1st step of prediction of CT1, CT2 should be entered, and vice versa.

**WE BELIEVE THAT OPERATING WITH TWO MEASURES OF CT ADDS STRENGTH TO OUR CLAIM THAT THERE EXISTS A GENERLISABLE TENDENCY TO ADOPT CTS IN MANY FORMS, AND THAT THIS GENERALISABLE TENDENCY IS LINKED TO THE PSYCHOLOGICAL MECHANISMS THAT WE OUTLINE.**

**THESE IDEAS ARE HOPFULLY NOW BETTER EXPLAINED IN THE TEXT**.

The other concern in this part is regarding stepwise regression analysis which is very criticized and should be avoided. Instead, please do a regular, enter regression. If there is a problem with multicollinearity you could try to merge some variables in composite scores if that is justified.

**THE STEPWISE REGRESSION IS NOW DELETED.**

- The other main concerns in regarding General Knowledge Test. It is not clear whether the authors shortened the test for this study or short version of the test is already validated elsewhere? Please report alpha for the used tests in the Method section and not in the Discussion. Furthermore, authors identify GKT with crystallized int. but the correlations between GKT with Openness are the same as with fluency test, while fluency test does not correlate sig. with Openness. I think that authors will need to be more careful regarding the conclusion about relations between crystall. int. and conspiracy theories since the used measure (GKT) and general knowledge as a construct per se is a construct between personality and intelligence. The conclusion should be more in line with what GKT really measures. Also, IQ in all tables and results should be replaced with GKT.

**WE HAVE DELETED REFEFENCES TO IQ AND REPLACED THEM WITH THE ABBREVIATION GK INSTEAD.**

- The joint factor analysis (p. 14) is not argumented in Introduction and it is not in line with aims. Please delete this part.

**DELETED AS SUGGESTED**

- In discussion, there is not much explanation of the results, for example, why low N and high C are associated with CT and not A and O as expected?

**THE DISCUSSION IS NOW RE-DESIGNED TO EXAMINE THE REGRESSION RESULTS THAT FOCUS ON THE PERSONALITY DISORDERS.**

Minor points:

- p.3 „Indeed, there are good theoretical reasons to believe that the other disorders (i.e. schizotypy) are more related to CTs than the two assessed in the dark triad [18].“ – please correct this because Dark Triad is subclinical and not clinical construct. The traits in DT are not considered disorders.

**REFERENCES TO THE DARK TRIAD ARE NOW TAKEN OUT OF THE CORE PART OF THE STUDY AND ONLY SPORADICALLY REFERRED TO AS PART OF THE PREVIOUS RESEARCH IN THE FIELD.**

**YOU AND THE REVIEWERS HAVE GIVEN US THE OPPORTUNITY TO SIGNIFICANTLY IMPROVE THE CLARITY, FOCUS AND ANALYSIS OF THE PAPER FOR WHICH WE ARE MOST GRATEFUL.**
