# Supplementary material for: Dispositional and ideological factor correlate of conspiracy thinking and beliefs
Source: PLoS One. 2022 Oct 26;17(10):e0273763. doi: 10.1371/journal.pone.0273763 (PMC9604007; doi:10.1371/journal.pone.0273763)
Supplement: Attachment — Submitted filename: PONE-D-21-40074R2 Comments to reviewers.docx [file pone.0273763.s003.docx]

***Comments to reviewers - PONE-D-21-40074R1***

***Dispositional and ideological factor correlate of conspiracy thinking and beliefs***

***PLOS ONE***

Dear prof. Knežević!

Please find below our comments and answers to the questions and concerns addressed by yourself and the reviewers:

The article is now considerably improved.

Thank you!

However, both reviewers had further recommendation that I would like you to address properly. While r#2 has more technical suggestions, the objections of r#1 are more general and substantive. The issue of importance and novelty of the findings in the context of existing knowledge should be more elaborated. As emphasized by both reviewers, the semantic analysis requires further explanation and elaborations. As none of us is familiar with the analysis (and probably most of our audience) the method and its rationale should be more elaborated.

The technique is now explicated in further detail at several points in the manuscript. The methodological approach has also been more aligned with traditional statistics tables. We have also added more references to the growing body of research using these methods. Hopefully these passages are more intuitively understandable now.

I am curious how you would respond to the following statement: to the extent that the relationship between any two constructs is based on the similarity of the words used to describe them, the more psychologically trivial is the relationship (i.e., constructs are too close to each other, predictor-criterion overlap is substantial).

This question is pertinent and interesting. However, it has been thoroughly dealt with in a number of the references in the text, these will be listed again here. The core of the problem is how almost all survey-based correlation matrices seem heavily influenced by semantic relationships. These semantic relationships seem to exist below the usual conventions for scale construction in psychometrics, such as various types of factor analysis, rotation of scales and thresholds for cross-loadings. Thus, several publications using semantic algorithms have shown that the predictor-criterion overlap problem even where the strictest psychometric techniques have been used. There is a growing need to determine the relationship between semantic properties of scales and the influence of attitude strength. In fact, the present manuscript offers an interesting window to the intricate relationship between attitudinal and semantic/cognitive mechanisms involved in survey responses, see table 6 which contains a direct comparison of semantic predicted with empirically observed relationships. The following publications address these questions directly:

The relationship between semantic patterns and attitude strength: Arnulf, J. K., Larsen, K. R., Martinsen, O. L., & Egeland, T. (2018). The failing measurement of attitudes: How semantic determinants of individual survey responses come to replace measures of attitude strength. *Behavior Research and Methods, 50(6),* 2345-2365.

The use of text algorithms to replace/complement Likert scales and other types of psychological measures: Kjell, O. N. E., Kjell, K., Garcia, D., & Sikstrom, S. (2019). Semantic Measures: Using Natural Language Processing to Measure, Differentiate, and Describe Psychological Constructs [Article]. *Psychological Methods*, *24*(1), 92-115. https://doi.org/10.1037/met0000191

The pervasiveness of semantically produced relationships in established measurement scales: Arnulf, J. K., Larsen, K. R., Martinsen, O. L., & Bong, C. H. (2014). Predicting survey responses: how and why semantics shape survey statistics on organizational behaviour. *PLoS ONE*, *9*(9), e106361. <https://doi.org/10.1371/journal.pone.0106361>

How semantic properties of scales may help us model scales and their relationships a priori: Rosenbusch, H., Wanders, F., & Pit, I. L. (2020). The Semantic Scale Network: An online tool to detect semantic overlap of psychological scales and prevent scale redundancies. *Psychological Methods*, *25*, 380–392. https://doi.org/10.1037/met0000244

The philosophical problems of semantically determined scales: Arnulf, J. K., & Larsen, K. R. (2021). Semantic and ontological structures of psychological attributes. In D. Wood, S. J. Read, P. D. Harms, & A. Slaughter (Eds.), *Measuring and modeling persons and situations* (pp. 69-102). Academic Press. https://doi.org/10.1016/B978-0-12-819200-9.00013-2

Apart from reviewers requested, I would suggest to have the values in the Table 6 better explained (Are correlations between PDS and CTs+GK presented in the last three columns?; How exactly sum factor scores from LSA are calculated and why this information is important beyond the three factors loadings?; Isn’t it the case that the correlations between the loadings on the three factors and correlations of PDs and CTs are of importance, not the correlations between the factor loadings and sum of loadings across the factors or their ranks).

Tables 5 and 6 are now completely changed in ways that comply more with traditional ways of representing data, and with more extensive explanations that we hope will make everything more transparent.

Please include the following items when submitting your revised manuscript:

- A rebuttal letter that responds to each point raised by the academic editor and reviewer(s). You should upload this letter as a separate file labeled 'Response to Reviewers'.

See below

- A marked-up copy of your manuscript that highlights changes made to the original version. You should upload this as a separate file labeled 'Revised Manuscript with Track Changes'.

See attached

- An unmarked version of your revised paper without tracked changes. You should upload this as a separate file labeled 'Manuscript'.

See attached

Reviewers' comments:

Reviewer's Responses to Questions

**Comments to the Author**

4. Have the authors made all data underlying the findings in their manuscript fully available?

requires authors to make all data underlying the findings described in their manuscript fully available without restriction, with rare exception (please refer to the Data Availability Statement in the manuscript PDF file). The data should be provided as part of the manuscript or its supporting information, or deposited to a public repository. For example, in addition to summary statistics, the data points behind means, medians and variance measures should be available. If there are restrictions on publicly sharing data—e.g. participant privacy or use of data from a third party—those must be specified.

All the necessary data will be uploaded with the manuscript

Reviewer #1: No

Reviewer #2: No

5. Is the manuscript presented in an intelligible fashion and written in standard English?

PLOS ONE does not copyedit accepted manuscripts, so the language in submitted articles must be clear, correct, and unambiguous. Any typographical or grammatical errors should be corrected at revision, so please note any specific errors here.

Reviewer #1: Yes

Reviewer #2: Yes

6. Review Comments to the Author

Reviewer #1: This revised paper focuses on a semantic analysis of various personality measures and tendencies toward conspiratorial thinking in a convenience sample of mostly British adults. The authors find that various clinical forms of thinking such as schizotypal tendencies are strong correlates of conspiracy thinking. This has been found before and so it seems that what is novel in this paper is the use of semantic analysis to identify why people who endorse those clinical symptoms are more likely to also believe in conspiracies. Publication in Plos One does not require novel findings, but if replication is the goal, then this is a poor sample for that purpose. It is limited to people who are employed, with no obvious justification for that restriction. And it is unlikely to be representative of any population since it is a convenience sample with no evidence of generalizability. The authors argue in the future directions section that the results raise interesting questions, but the ones suggested seem to have been studied. There is evidence that conspiracy believers use non-mainstream media and that they are attracted to alternative medical treatments. All of this prior work should be cited and if the present results add something of value to those findings, then it might be worth saying what that is.

We believe that our sample of nearly 400 adults drawn from the Prolific platform is a good sample from which one could easily generalize. Indeed, we have published papers in PLOS ONE (2021) using a similar sample from the same provider. We specify employed people to reduce the number of students.

As to the latter point, we have noted the salient prior work relevant to this paper and tried to point out where we are adding something new

With these concerns aside, there are questions about how the semantic analysis was conducted. How were the inter-relations between the semantic scores subjected to a principal component analysis? I can imagine how that was done, but providing the details would help.

These details are now provided with more elaborate explanations.

The more important analysis of the relation between the semantic scores and conspiracy beliefs is totally opaque. It is not at all clear what is meant by the sum of factor loadings in Table 6 or how those were related to ratings of conspiracy beliefs. Nor is there any clear interpretation of the second factor that is claimed to correlate with conspiracy thinking.

Hopefully this is now easier to understand. The semantic analyses build on a widely published framework, now extensively cited and explained in the text. The techniques are explained and compared with the empirically obtained survey responses. In the most important table, number 6, we show that the semantic and empirical relationships are correlated .70 (p < .01) which hardly suggests a serendipitous result. The table should make it clear that the cognitive structures of the survey item texts alone show strongest relationships between cluster A scales and conspiration theories, and this pattern is detectable in the respondent data.

The authors seem to gloss over the finding that conscientiousness is related to conspiracy thinking. Why is that? Given the unequal reliabilities of the various measures used to assess correlates of conspiracy thinking, regression analysis is a poor guide to detecting true relations. I would go with the simple correlations more so than the regressions.

We have noted and discussed the conscientiousness finding. We have kept in the regressions recognizing that the problems highlighted by the reviewer are very common in this area.

The authors claim that the two measures of conspiracy mentality are only modestly correlated (.58) but given the reliabilities of the measures of .60 and .91 (as reported in the Discussion when it should be in the Methods), one would only expect a correlation of about .54. So, the correlation that was observed suggests that they two scales are virtually identical.

We have changed the “modestly” correlated to “strongly” correlated though we cannot accept that they are virtually identical.

Reviewer #2: Dear authors,
Thank you for your answers and comments. The manuscript is improved, but there are still some minor issues that should be addressed before the acceptance for publication:

Introduction
- Please add a short explanation of similarities and differences between 2 measures of CTs since you built your case based on these 2 measures.

See above…we have done this.

- Seems like the whole introduction is based on belief in CTs, and not conspiracy thinking. In the description of previous research please specify for each study which CTs measure is used
(specify the name of the measure, i.e., in Walter and Drochon study, etc.). This would contribute to a better understanding of the introduction of 2 measures of CT.

We have tried to do that. We have also tried to explain similarities and differences between Belief if CTs and conspiracy thinking, and further we have changed the title into “…conspiracy thinking and beliefs” to reflect this.

- Although the authors stated that they delete the term cryst. int., it is still present in the hypothesis: "We also used a short measure of crystalised intelligence to test the hypothesis that higher IQ test scores would be negatively correlated with belief in CTs (H2)." as well as in the whole text (e.g., in the discussion they used the term intelligence and not cryst. int.).

Now done

Please specify that you expect negative relations between the general knowledge as the indicator of cryst. int. and CTs, or just - negative relations between the general knowledge and CTs.

The latter…now done

In addition, in this hypothesis, only beliefs in CTs are mentioned and not conspiracy thinking or CTs in general, as in other hypotheses.

OK…rectified to be consistent

Results
- There is no M and SD for CT2 in Table 1, please merge Tables 1 and 3. Please add alphas for all measures in Table 1 and report either sum or mean scores for used instruments (mean scores are better). Please delete descriptives for sex, it is odd. Add a note with variable explanation in all tables.

Done as requested

- I am not sure why in LSA only CT1 was entered, and not CT2. Please explain it at the beginning of the analysis. Are there some technical issues? I am not familiar with LSA and I could assume that readers would not be also familiar, thus the comment "(CT2 was not part of the analysis at all and the number is statistically significant even if N (the number of scales) is only 14)" should be explained more clearly.

This passage is now changed. The reason CT2 is taken out is that long matrices of single items can get unwieldy in LSA, since the number of unique item pairs expands with every item added. We believe that the statistics and whole picture is easier to understand using only CT1, since the two are virtually interchangeable in effects, as per the comments of one of the reviewers.

- Seems like the rotation was not used in PCA, was it on purpose? Also, the statement "Here, the CT1 items have the strongest loadings, followed by the items from schizotypal scale" is not true, since the Depressive scale had loading -.216.

To change the presentation of data, we have now shown a factor structure of the SCATI and CT1 based on a PCA with oblimin rotation. The ensuing picture is clearer. Negative loadings are tricky from an interpretive point of view in semantics and we have now tried to make the display of data more aligned with usual statistical representations.

- The following analysis presented in Table 6 is not clear to me and it is not clear why GK is in the table. The answer on your H9 could be get based on the pattern structure of the factors in Table 5 (with the use of rotation). If you have other rationale, please explain. Also sentence "If the CT1 factor loadings are removed from the table..." should be changed to "removed from the analysis..."

This is now all changed.

Discussion
- The discussion lack similarities and differences between CT1 and CT2. In addition, the statement "In this study we measured a number of individual variables which accounted for
22% of the variance in both analyses" should be replaced with - around 20%, since CT1 was explained by 20% and CT2 by 21%.

Now changed thank you.

Other
- Check APA for use of capital letters (it should be personality disorder and conspiracy theories, but Dark Triad and not dark-triad or dark triad)

Done as requested

- Delete (Crystallised IQ) from the explanation of the General Knowledge Test.

Done as requested

- Delete M and SD from the description of the measures since they are already presented in Table 1.

Done as requested

- Results for model testing remove from Table 2 (Model Adj. R2 = .20. F = 6.868, p < .001) and Table 4 (Model Adj. R2 = .21. F = 7.250, p < .001) and report in text as you did for next analysis.

Done as requested

- Please specify on p.10 that you use hierarchical regression analysis.

Done as requested

**We very much hope that you can accept this revision for the journal**
